# Supplementary material for: The Systems Biology Research Tool: evolvable open-source software
Source: BMC Syst Biol. 2008 Jun 29;2:55. doi: 10.1186/1752-0509-2-55 (PMC2446383; doi:10.1186/1752-0509-2-55)
Supplement: Additional file 1 — SBRT Archive. An archive of the current version of the Systems Biology Research Tool. [file 1752-0509-2-55-S1.zip › sbrt-1.4.0/doc/developers_guide/api/sbrt/kernel/fba/proc/FbaProcess.html]

FbaProcess


|  |  |  |
| --- | --- | --- |
| **Overview** \| **Package** \| **Class** \| **Use** \| **Tree** \| **Deprecated** \| **Index** \| **Help** | |  |
| **PREV CLASS**   **NEXT CLASS** | **FRAMES**    **NO FRAMES**     **All Classes** |  |
| SUMMARY: NESTED \| FIELD \| CONSTR \| METHOD | DETAIL: FIELD \| CONSTR \| METHOD |  |


---


## sbrt.kernel.fba.proc Class FbaProcess<R>

```
java.lang.Object
  sbrt.kernel.fba.proc.FbaProcess<R>
```

**Type Parameters:**: `R` - the results type.

**All Implemented Interfaces:**: KernelProcess<R>

**Direct Known Subclasses:**: CatalystDeletionProcess, CdHarFluxVectorProcess, CvCatalystDeletionProcess, CvFbaOptimizationExperiment, CvFbaOptProcess, CvObjectiveFunctionProcess, CvRxnDeletionProcess, CycleIdProcess, ExtremeCurrentProcess, FbaOptimizationProcess, FbaSolverProcess, FluxActivityProcess, FluxCapProcess, FluxIntervalProcess, FluxomeReducerProcess, FluxPlasticityProcess, InitialPointGenerator, NetworkInfoProcess, ObjectiveFunctionAnalysis, RandomConstraintsGeneratorV1, RandomEnvGeneratorV1, RandomObjectiveGenerator, RxnDeletionProcess, StoichVariationProcess

---

``` public abstract class FbaProcess<R> extends java.lang.Object implements KernelProcess<R> ```

This abstract class is used to represent Flux Balance Analysis experiments.

**Author:**
:   This class was written and documented by Jeremiah Wright while in
    the Wagner lab.

---

**Constructor Summary**

`FbaProcess()`


| **Method Summary** | |
| --- | --- |
| `abstract  Fluxome` | `getFluxome()`             Returns the fluxome used in this experiment. |
| `java.lang.String` | `toString()`             Returns a string representation of this experiment. |

**Methods inherited from class java.lang.Object**

`clone, equals, finalize, getClass, hashCode, notify, notifyAll, wait, wait, wait`

**Methods inherited from interface sbrt.kernel.KernelProcess**

`run`

**Constructor Detail**

### FbaProcess

```
public FbaProcess()
```


**Method Detail**

### getFluxome

```
public abstract Fluxome getFluxome()
```

:   Returns the fluxome used in this experiment.

    :   **Returns:**: the fluxome used in this experiment.

---


### toString

```
public java.lang.String toString()
```

:   Returns a string representation of this experiment.

    :   **Overrides:**: `toString` in class `java.lang.Object`
    :   **Returns:**: a string representation of this experiment.


---


|  |  |  |
| --- | --- | --- |
| **Overview** \| **Package** \| **Class** \| **Use** \| **Tree** \| **Deprecated** \| **Index** \| **Help** | |  |
| **PREV CLASS**   **NEXT CLASS** | **FRAMES**    **NO FRAMES**     **All Classes** |  |
| SUMMARY: NESTED \| FIELD \| CONSTR \| METHOD | DETAIL: FIELD \| CONSTR \| METHOD |  |


---
